# Highly Modulated In-Fiber Mach–Zehnder Interferometer Based on an Ultracompact Leaky-Guided Liquid Core

**DOI:** 10.3390/s22030808

**Published:** 2022-01-21

**Authors:** Cheng-Ling Lee, Wei-Rong Zhuo, Tai-Kai Liu

**Affiliations:** Department of Electro-Optical Engineering, National United University, Miaoli 360, Taiwan; c0974022750@gmail.com (W.-R.Z.); z8008280082@gmail.com (T.-K.L.)

**Keywords:** Fiber Mach–Zehnder interferometer (FMZI), fiber-optic sensor, leaky-guided liquid core (LLC), hollow-core fiber (HCF), thermal optics coefficient (TOC)

## Abstract

We proposed a novel sensor based on an ultracompact leaky-guided liquid core fiber Mach–Zehnder interferometer (LLCFMZI) for high modulation of an interference spectrum. The sensor structure is based on a micro-sized hollow-core fiber (HCF) splicing a tilt end face single-mode fiber (SMF) to create a miniature oblique gap for the effective access of different liquids. The liquid core with a relatively lower refractive index (RI) than the cladding can achieve a leaky-mode optical waveguide (LMOW) mechanism, and its volume is only approximately 7.85 pL. In addition, the utilized micro-length HCF can reduce the energy loss of core in the LMOW to obtain an acceptable extinction ratio (>30 dB) with high temperature (T) sensitivity in the interference spectra. Experimental results show that the interference spectra can be highly modulated within the wide measurement range of 1250–1650 nm with a steadily linear response for thermal effect. The measured temperature sensitivities (T-sensitivities) of various liquids of DI water, ethanol, and Cargille-liquid (n_D_ = 1.305) are 0.8869, 4.4754, and 4.8229 nm/°C, and the corresponding measured thermal optics coefficient (TOC) are −4.16 × 10^−5^, −2.11 × 10^−4^, and −3.6 × 10^−4^ °C^−1^, respectively. Measurement results demonstrate that the used liquids with a higher TOC can obtain better T-sensitivity modulation. The highest experimental sensitivity of the liquid-core filled with Cargille-liquid (n_D_ = 1.40) is up to +13.87 nm/°C with a corresponding TOC of −4.07 × 10^−4^ °C^−1^. Furthermore, the experimental and theoretical values are in good agreement according to FSR the measuring scheme that investigates the effectiveness of the proposed LLCFMZI.

## 1. Introduction

Fused silica fiber has intrinsic properties of low thermal optics coefficient (TOC), low thermal expansion coefficient (TEC), and high stiffness with low tensile (i.e., high Young’s modulus), such that it is not particularly sensitive to some sensing parameters. To achieve high sensitivity, numerous optical fiber sensors are combined with other materials by utilizing dispersion features to control the energy of the core and cladding in the optical waveguide, an approach which significantly changes the optical characteristics of the propagating core and cladding modes. Optical fiber Mach–Zehnder interferometers (FMZIs) based on the interference of the core and cladding modes can achieve superior interference characteristics, because their interfering modes have the potential of being highly tunable. Consequently, extensive research combines various materials with photonic crystal fiber [1,2], hollow-core fiber (HCF) [3,4], twin-core fiber [5], and D-shaped fiber [6] for the fabricated FMZIs. The silica-based fibers combined with the other materials, including liquids [4,5,6,7,8,9,10,11,12], nanoparticles [13,14], and 2D materials (graphene) [15,16,17,18,19], would generate much higher sensitivity to external parameters than that of the intrinsic silica fibers. Various configurations for the FMZIs have been proposed in the literature and have been demonstrated their effectiveness [1,2,3,4,5,6,7,8,9,12,13,14,15,16,17,18,19,20,21,22,23,24,25,26,27,28]. Examples include an FMZI with anti-resonance based on an multi-mode fiber (MMF)-HCF-MMF structure for sensing application [21], tapered fiber-based FMZI for the refractive index (RI) and temperature (T) measurement [22], core-offset in-line FMZI [23], an in-line FMZI with thin-core fiber (TCF)-thin fiber-TCF [24], and SMF-HCF-SMF structures [12,20,28,29]. The structures of the above FMZIs mainly create two arms of the interferometer: The core of the fiber waveguide and the cladding which propagates one or more cladding modes. Furthermore, many FMZIs combined with other materials can possibly promote more flexible applications for specific functionalities, thus numerous types of research have been conducted to exploit all kinds of fabricating technology and structures for advanced FMZIs. In 2017, an FMZI structure based on the HCF coated inner zinc oxide film was developed to improve the output optical spectrum [29]. Another FMZI sensor with a tungsten disulfide-coated surface was intended for measuring ethanol vapor concentration [25], high TOC and large elasticity PDMS sealed a micro-FMZI for the simultaneous measurement of pressure and T [26], and a single core-offset FMZI coated with polyvinyl alcohol was designed for the simultaneous measurement of relative humidity and T [27].

Given the high modulation of RIs in the liquids, combining optical fiber has a broad range of applications. The application of fiber-optic sensors combined with liquids entails changing the effective RI of the external surroundings [8], cladding modes [6,7,9], or core mode [4,12] for the FMZIs. In a section on changing the external surroundings of the fiber, the study of Zhu et al. reported an FMZI based on tapered hollow optical fiber. They immersed the FMZI sensor into the water–glycerin to investigate the characteristics of the surrounding RI responses [8], and this method consumed a considerable amount of liquids. In 2012, Kieu et al. used a gap-splice to easily introduce liquid CS_2_ of high RI into a 1 m length of HCF for a nonlinear optical experiment of Raman scattering [10]. Liu et al. proposed a simple fiber sensor for simultaneous measurements on RI and T. The fiber sensor consists of a section of liquid-filled HCF with the length of a few centimeters spliced between SMFs. The super-high T of the fusion splicing by arc-discharging after filling with liquid makes the liquid almost volatilize in the fiber core, thereby resulting in failed splicing [12]. The structure thus fails to achieve the effect of high sensitivity. Therefore, the structure with the liquid core fiber can be ameliorated to combine with dispersion engineering so that the optical waveguide characteristics of FMZI can be changed. The RIs of the liquids which are introduced into the core is usually a little higher than that of the cladding to achieve a guided mode in the optical fibers. However, Jung et al. first reported an ultracompact in-line FMZI which is generated by a leaky waveguide in the air hollow-core fiber spliced between two SMFs [28]. As the effective RI of the core mode is much lower than that of the cladding mode, a tiny HCF can ensure that the energy of the core does not completely leak out for attaining an acceptable FMZI performance. From the previous reference, it can be inferred that liquids with low RIs can be utilized to form the leaky-guided liquid core (LLC) fiber and fabricate a leaky-guided liquid core fiber Mach–Zehnder interferometer (LLCFMZI) based on the leaky-mode optical waveguide (LMOW) mechanism. Given the LMOW mechanism, the RI of the liquid-core is relatively lower than that of the cladding and using a micro-sized HCF is necessary to generate the MZI, by maintaining comparable energy between the core and cladding modes before the light in the leaky-core completely dissipates. The long HCF of over 2 cm [12] and 6~8 mm [20,29] lacks special structures that prevent effective filling in by liquids, which means the above FMZIs have drawbacks of excessive use of liquids, liquid evaporation by fusion splicing, insensitivity, excessive energy dissipation, or poor interference visibility.

In this study, we proposed an innovative sensor which is a highly sensitive in-fiber Mach–Zehnder interferometer based on an ultracompact leaky-guided liquid core. The structure of the sensor is based on a very tiny section of HCF splicing a tilt endface SMF to create a miniature oblique gap for the easy access by liquids. The utilized micro-length liquid core fiber efficiently reduces the energy loss in the LMOW mechanism, as well as obtains a good extinction ratio (>30 dB) and high T sensitivity in the interference spectra. Note that the filled liquid with low RI can achieve the LMOW mechanism and its volume is only approximately 7.85 pL. Experimental results show that the interference spectra of LLCFMZI can have highly modulated characteristics within a wide wavelength range of 1250–1650 nm, with a steadily linear response for thermal modulation. Regarding the quality of the interference spectra, fringe visibility over 30 dB is achieved. Measurement results demonstrate that the used liquids with higher TOC values can obtain better T-sensitivities. Therefore, the design of the proposed LLCFMZI would be more flexible by choosing different liquids for specific applications. The highest experimental sensitivity of the liquid core filled with Cargille-liquid (n_D_ = 1.40) is up to 13.87 nm/°C and its corresponding TOC is −4.07 × 10^−4^ °C^−1^. Furthermore, the experimental values are in good agreement with the theoretical values for investigating the effectiveness of the present measuring scheme.

## 2. Fabrication and Principle

In the experiment, we first fusion spliced the SMF with a section of 100 μm length (L) HCF whose inner core and outer cladding were 10 and 125 μm, respectively. Subsequently, we utilized slant cleaving on the other SMF endface (by Fujikura CT-100) and splicing on the HCF to generate a tiny gap between the HCF and SMF (by Fujikura FSM-100M+) for ensuring easy access for the liquid. In general, the use of laser drilling is an effective method to create a microhole on the HCF. However, the cladding thickness of the used HCF was relatively thick (57.5 μm), thus laser drilling was not a good method to create a microhole. Thus, by using oblique cleaving and splicing fusion techniques under controlled parameters, the developed method has the advantages of cost-effectiveness, easy fabrication, and convenience.

**Figure 1:**
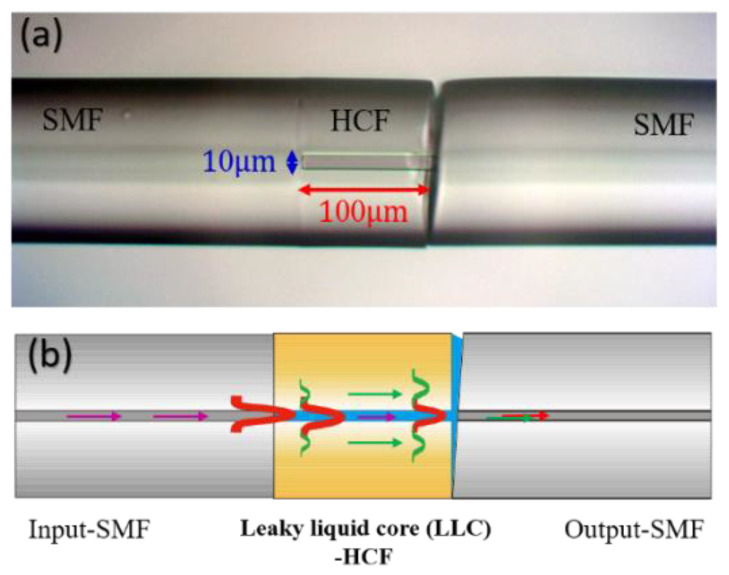
(**a**) Microphotograph of the LLCFMZI sensor. The LLC-HCF with D = 10 μm and L~100 μm. (**b**) Schematic of the interference between the leaky-core and cladding modes in the LLCFMZI.

The other advantage of the tilt angle of the SMF is the prevention of the collapse of the hollow core by the fusion splicing. Note that the oblique angle is approximately 8° and can resist the undesired reflected noise caused by the Fresnel reflection of light. Finally, we employed the capillary phenomenon to fill a drop amount of liquid into the hollow core through the tiny gap. In the study, the RI of the liquid-core was smaller than that of the silica cladding for obtaining a leaky core mode. Figure 1a shows the microphotograph of a micro-sized LLCFMZI sensor for achieving high sensitivity and the measurement of the TOC of the liquids. Figure 1b plots the main region, leaky-guided liquid core-hollow core fiber (LLC-HCF) splits the leaky core and cladding modes for the interference.

**Figure 2:**
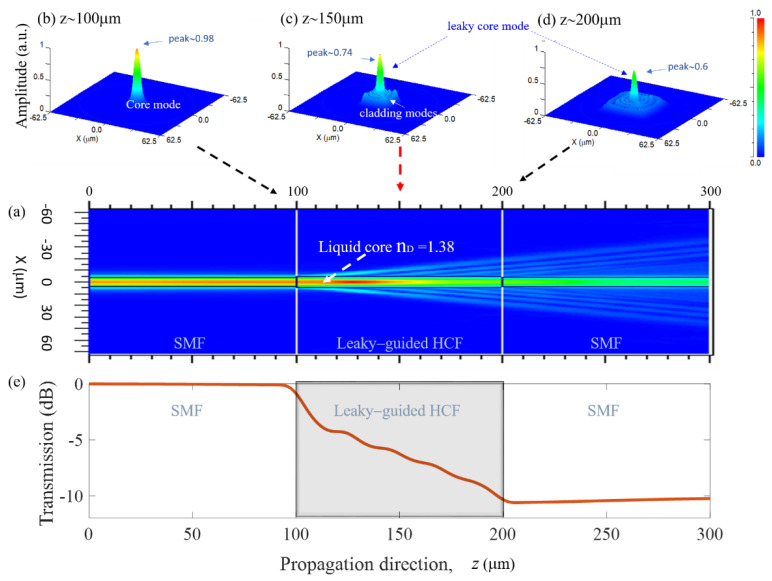
(**a**) FDBPM simulation of (**a**) optical light propagation and (**b**–**d**) evolution of the 3D optical power distribution inside the device configuration at (**b**) *z* = 100 μm, (**c**) *z* = 150 μm and (**d**) *z* = 200 μm, respectively, and (**e**) optical transmission of the light propagation through the LLCFMZI.

The proposed LLCFMZI was mainly formed in the HCF region and its interference spectra was analyzed using an optical spectrum analyzer (OSA). However, given the optical characteristics of the LLCFMZI achieved by the LMOW, the optical light from the SMF core would gradually leak out into the silica cladding through the increasing propagation distance and enhanced optical light attenuation (Figure 2). Figure 2a shows the optical power of modes propagating inside a leaky-liquid HCF with L = 100 μm, D = 10 μm, and RI of liquid core n_D_ = 1.38, which was calculated by the finite difference beam propagation method (FDBPM) of the numerical software Rsoft^®^Beam PROP when the input wavelength is λ = 1550 nm. Clearly, the filler liquid whose RI is lower than that of the fiber cladding causes the core light to gradually leak out into cladding along the HCF. Figure 2a shows the normalized amplitude of optical power that propagates along the *z* axis inside the leaky-guided liquid-core HCF and the 3D optical power distribution are shown in Figure 2b–d. Figure 2b–d shows the simulation results of the power distribution in the LLCFMZI at *z* = 100, 150, and 200 μm, respectively. A core mode from input-SMF in Figure 2b clearly leaks out into the cladding to induce cladding modes in Figure 2c,d and reduces the peak power of the core mode. The leaky core mode and the induced cladding mode were formed two optical paths in the fiber structure and expected for achieving the Mach–Zehnder interference. Moreover, we can see that the optical transmission of the core mode in the LLCFMZI is shown in Figure 2e. The transmission was monitored at the central optical axis *x* = 0 propagating along the *z*-axis. The result investigates the optical power of light traveling through the leaky HCF that suffered a leaky loss. Increasing propagation distance of HCF would enhance the optical power attenuation in the core. We posit that the LLCFMZI with a longer HCF can result in a higher leaky loss along the *z*-axis direction. The simulation results of Figure 2e indicate that the transmission loss about −10–−11 dB is obtained in the case of the HCF with L = 100 μm.

The micro-sized HCF is tiny enough not to make light of the fiber core dissipate completely. The remaining light of the core and one of the cladding modes can overlap in the output-SMF to generate interference. The basic principle of the LLCFMZI is the input beam is gradually dispersed into two portions along the HCF, and the two beams will respectively propagate through silica cladding and the leaky liquid-core. Finally, the cladding and the core beam will recombine at the second junction and generate interference. The optical intensity of cladding and core beams in the LLCFMZI are, respectively, denoted as I__clad_ and I__core_, and the total intensity of the interference beam, denoted as I, is defined by Equation (1).

(1) $$I=I_{\mathrm{core}}+I_{\mathrm{clad}}+2\sqrt{I_{\mathrm{co}}\cdot I_{\mathrm{cl}}}\cos\left(\frac{2\pi }{\lambda }{\Delta n}^{\mathrm{eff}}L\right)$$

where ${\Delta n}^{\mathrm{eff}}=n_{\mathrm{cl}}^{\mathrm{eff}}-n_{\mathrm{co}}^{\mathrm{eff}}$ is the effective index difference between cladding mode and core mode. λ is main wavelength of the light. When the optical phase difference ((2π/λ)⋅Δn^eff^⋅L) conforms the condition of destructive interference, the dip wavelength of minimum power ($\lambda_{\min}^{m}$) can be deduced as Equation (2).

(2) $$\lambda_{\min}^{m}=\frac{2}{2m+1}{\Delta n}^{\mathrm{eff}}L$$

Here, m is the order of interferential mode and it is an integer. Free spectral range (FSR) of the FMZI means that the wavelength difference of two continuous interference dips or peaks between front and rear. Afterwards, we can utilize the relationship of interference phase difference to derive FSR, which can be expressed as Equation (3).

(3) $$\mathrm{FSR}=\left|\lambda_{1}-\lambda_{2}\right|=\frac{\lambda_{1}\lambda_{2}}{\left|n_{\mathrm{cl}}^{\mathrm{eff}}-n_{\mathrm{co}}^{\mathrm{eff}}\right|L}$$

where λ_1_ and λ_2_ represent the wavelengths of two adjacent interference dips. $n_{\mathrm{cl}}^{\mathrm{eff}}$ and $n_{\mathrm{co}}^{\mathrm{eff}}$, respectively, are the effective refractive index of cladding and core modes for the interference. Turning now to the thermal effect on the interference spectra. λ and λ^′^ corresponds to two wavelengths of interference dips at specific T and the T^′^ after heating up, respectively. There is an identical destructive phase at λ and λ^′^. The FSR is approximately the same as it was before after heating up. Consequently, we can calculate the following by Equation (4).

(4) $$\frac{\left(n_{\mathrm{co}}^{\mathrm{eff}}-n_{\mathrm{cl}}^{\mathrm{eff}}\right)\times L}{\lambda }=\frac{\left({\;n\prime }_{\mathrm{co}}^{\mathrm{eff}}-{\;n\prime }_{\mathrm{cl}}^{\mathrm{eff}}\right)\times \;L\prime }{\;\lambda \prime }$$

where ${\;n\prime }_{\mathrm{co}}^{\mathrm{eff}}$,${\;n}^{\prime }{}_{\mathrm{cl}}^{\mathrm{eff}}$ and L′ are the parameters after heating. The TEC of silica ($\alpha =5.5\times 10^{-7}\;{℃}^{-1}$) is an extremely small scale compared with other parameters [30]. Therefore, the length of HCF is almost the same before and after heating. Equation (4) can be further deduced through the concept of the TOC and to be reorganized into Equation (5) to calculate the TOC of the liquid-core (k_co_).

(5) $$k_{\mathrm{co}}=\frac{\left(\frac{\;\lambda \prime }{\lambda }-1\right)\times \left(n_{\mathrm{co}}^{\mathrm{eff}}-n_{\mathrm{cl}}^{\mathrm{eff}}\right)+\Delta T\times n_{\mathrm{cl}}^{\mathrm{eff}}\times k_{\mathrm{cl}}}{\Delta T\times n_{\mathrm{co}}^{\mathrm{eff}}}$$

Here, k_cl_ is TOC for the cladding of fiber (TOC =$\;\mathrm{TOC}=10.5\times 10^{-6\;}{℃}^{-1}$). ΔT denotes the T variation. Owing to the RI of liquid being highly related to the T, the high TOC of the materials can be predicted to have high temperature sensitivity (T-sensitivity). The wavelength of m-order ($\lambda_{\min}^{m}$) interference dips can be obtained from Equation (2). Next, the T-sensitivity: S (S ≡ $\frac{d\lambda }{\mathrm{dT}}$) for the LLCFMZI can be easily calculated as Equation (6).

(6) $$\frac{d\lambda }{\mathrm{dT}}=\lambda \left(\frac{k_{\mathrm{cl}}\cdot n_{\mathrm{cl}}^{\mathrm{eff}}\left(T\right)-k_{\mathrm{co}}\cdot n_{\mathrm{co}}^{\mathrm{eff}}\left(T\right)}{n_{\mathrm{cl}}^{\mathrm{eff}}\left(T\right)-n_{\mathrm{co}}^{\mathrm{eff}}\left(T\right)}\right)$$

where $n_{\mathrm{cl}}^{\mathrm{eff}}$(T) and $n_{\mathrm{co}}^{\mathrm{eff}}$(T) are the effective index of silica cladding and liquid core at a specific T, respectively. Based on the theoretical relation of Equation (6), we learn that the T-sensitivity of LLCFMZI increases as RI of liquid is close to the RI of cladding. In terms of value for Equation (6), ($n_{\mathrm{cl}}^{\mathrm{eff}}\left(T\right)-n_{\mathrm{co}}^{\mathrm{eff}}\left(T\right)$) is positive and k_co_ is generally negative. Thus, the values of the S are positive. That means that the interference spectra shift to longer wavelengths when T of the device increases.

## 3. Experimental Results and Discussion

**Figure 3:**
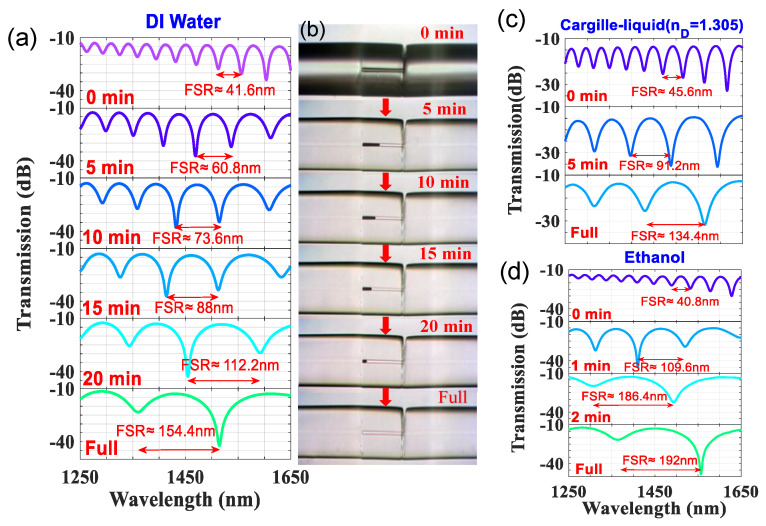
Evolutions of (**a**) duration corresponding spectra and (**b**) microphotographs of the DI-water-filled into the proposed LLCFMZI. Optical spectra evolution for filling (**c**) Cargille-liquid (n_D_ = 1.305) and (**d**) ethanol, respectively.

To study how the liquids fill into the HCF, several liquids with different properties were utilized. Since the length of HCF is fixed at 100 μm, which is so tiny, only one slant SMF endface splicing one side of HCF, to create the tiny gap for the liquid access, was required. The interaction force between the liquid and inner core and the duration of the liquid’s filling are highly correlated. Adhesive force and surface tension (ST) of liquid molecular generate a capillary phenomenon inside the hollow core. It makes liquid naturally fill into the hollow core by capillary action. Because of greater ST, the flow velocity is slower. The ST of DI water, ethanol, and Cargille-liquid were n_D_ = 1.305 are 72.67 (mN/m) [30], 22.30 (mN/m) [30] and 18 (mN/m) [31], respectively. Thus, the DI water with the highest ST which is filled in takes the longest time. On the other hand, the values of ST of the ethanol and Cargille liquid are similar, so we should also consider the viscosity of the liquids. The corresponding viscosities of these liquids are 1.005 (cP) [31], 1.18 (cP) [31], and 28 (cP) [32] for the DI water, ethanol, and Cargille liquids, respectively. It can be analyzed that the ethanol with small ST also has low viscosity and can take the shortest duration to fully fill the HCF. Figure 3 shows the performances of different liquids filled into the LLCFMZI. In Figure 3a the evolution of the duration corresponding to the interference spectra are shown, and in Figure 3b the microphotographs of the DI water gradually filled into LLCFMZI are displayed. We can find that the FSR gradually becomes wider as the liquid-filled area increases in the hollow core. It can be easily realized by an understanding of Equation (3). Because the optical path of the core ($n_{\mathrm{co}}^{\mathrm{eff}}$·L) in HCF steadily increases during the filling, the FSR is getting broader until completely filled. When completely filled, the optical spectra will maintain stability and do not change.

**Figure 4:**
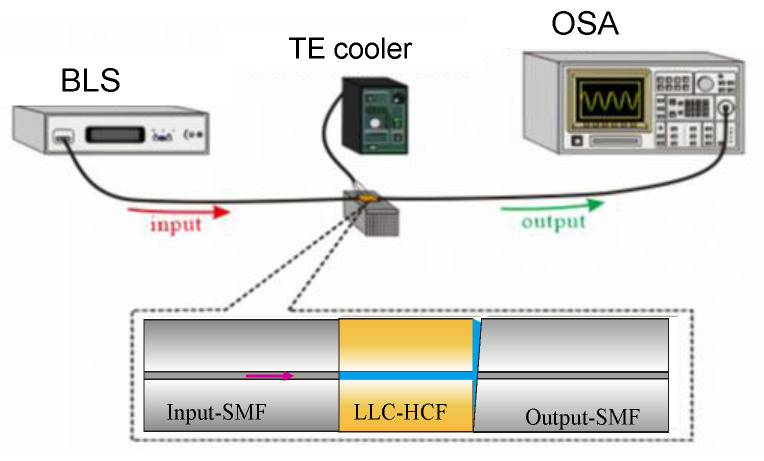
The schematic diagram of the measured system for the LLCFMZI.

After these devices were fabricated, their T was controlled exactly by using a TE cooler (resolution: 0.05 °C) to investigate the thermal effect of the LLCFMZI. The relevant experimental setup for making the measurement is displayed in Figure 4. It can be observed that the measurement system is quite simple. When a broadband light source (BLS) with wavelengths of 1250~1650 nm is incident from input-SMF to the LLC-HCF, it induced the cladding mode and interfered with the leaky-guided core mode. Afterward, the interference spectra could be readily measured by an optical spectrum analyzer (OSA).

**Figure 5:**
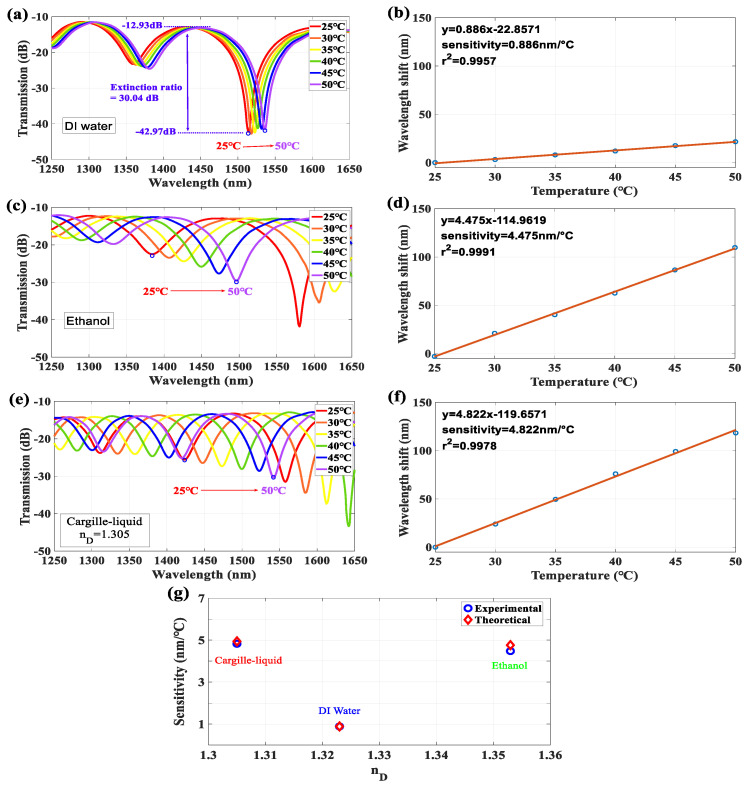
Interference spectra corresponding to the T variation for the filled liquids of (**a**) DI water, (**c**) ethanol, and (**e**) Cargille-liquid (n_D_ = 1.305). (**b**,**d**,**f**) Show the corresponding T-sensitivity. (**g**) Sensitivities of the experimental and theoretical values for filling the above liquids.

We varied T of the LLCFMZI from 25 to 50 °C with an interval of 5 °C to evaluate the thermal effect on interference spectra, as shown in Figure 5. The T-sensitivity of wavelength shift (Δλ) is high and positive, as pointed out in the principle on Equation (6), which demonstrates that the experimental results are consistent with the previous analysis for the spectra shift to a longer wavelength side when the T increases. The optical spectra corresponding to T variation are shown in Figure 5a,c,e for filled liquids of DI water, ethanol, and Cargille-liquid (n_D_ = 1.305), respectively.

The measured T-sensitivity exhibits extremely linear responses and steady characteristics. It is worth noting that the extinction ratio would be over 30 dB and insertion loss is around −13 dB for the HCF filled with these liquids, as shown in Figure 5. The result indicates that the interference visibility is much more significant than other structures of fiber-based interferometers. In Figure 5b,d,f, the corresponding T-sensitivity of filling with DI water, ethanol, and Cargille-liquid (n_D_ = 1.305) are measured, with 0.8869, 4.4754, and 4.8229 nm/°C, respectively. From Equation (6), one can calculate the correspondingly theoretical T-sensitivity of 0.8859, 4.7582, and 4.9380 nm/°C for the DI water, ethanol and Cargille-liquid (n_D_ = 1.305), respectively. Here, the parameters of $n_{\mathrm{cl}}^{\mathrm{eff}}$ (25 °C, 1550 nm) are set at 1.444 for the lowest-order cladding-mode (LP_02_) and the LP_01_$,{\;n}_{\mathrm{co}}^{\mathrm{eff}}$ (25 °C, 1550 nm) would be a complex number that depends on the RI of filled liquids. The values are 1.321, 1.352, and 1.299 for the DI water, ethanol, and Cargille-liquid (n_D_ = 1.305), respectively, and are substituted into Equation (6) for the theoretical calculation. The comparison of the T-sensitivities between experimental and theoretical values is illustrated in Figure 5g. Subsequently, we utilized Equation (5) and experimental parameters to estimate the TOCs of the measured liquids. The obtained TOCs of DI water, ethanol, and Cargille-liquid are −4.16 × 10^−5^, −2.11 × 10^−4^, and −3.6 × 10^−4^ °C^−1^, respectively. The above-determined TOCs calculated by Equation (5) were further compared with those in the literature and the results showed the consistency, as in Table 1. The above results also reveal that liquids with a larger TOC can have higher T-sensitivity in the LLCFMZI.

**Figure 6:**
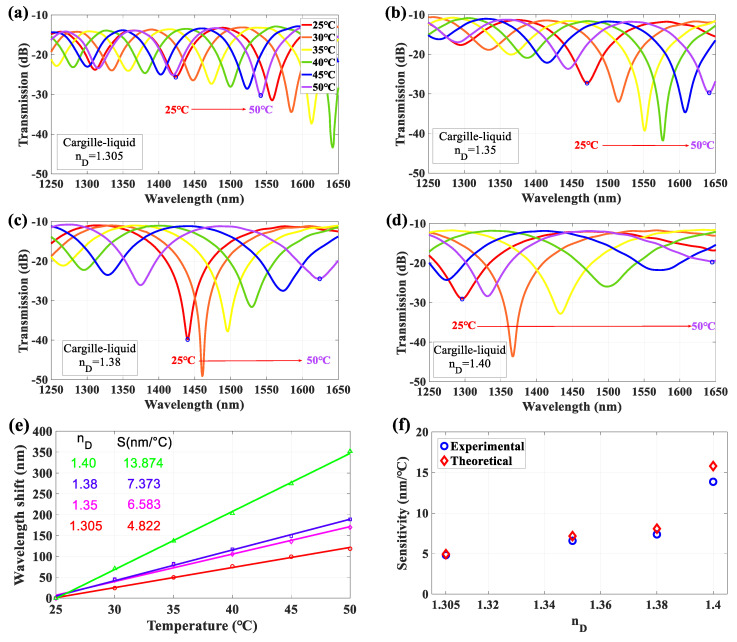
Measured interference spectra with different T values for the LLCFMZI filled with Cargille-liquids of n_D_ = (**a**) 1.305, (**b**) 1.35, (**c**) 1.38, and (**d**) 1.40. (**e**) T-sensitivities of Δλ for different Cargille-liquids. (**f**) T-sensitivities of the experimental and theoretical values for different Cargille-liquids as fillers.

For investigating the thermal effect of LLCFMZI under the conditions of liquid-core with different RI, several Cargille-liquids (standard RI liquids) were tested to evaluate the T-sensitivity and obtain the TOCs. Figure 6a–d shows the measured interference spectra with T variation for the Cargille-liquids of n_D_ = 1.305, 1.35, 1.38, and 1.40, respectively. The corresponding experimental T-sensitivities are 4.823, 6.583, 7.374, and 13.874 nm/°C for n_D_ = 1.305, 1.35, 1.38, and 1.40, respectively, as plotted in Figure 6e.

Similarly, as previously calculated by Equation (6), the comparison of T-sensitivity between experimental and theoretical values is shown in Figure 6f. Here, the theoretical values are calculated as 4.938, 7.167, 8.081, and 15.814 nm/°C for n_D_ = 1.305, 1.35, 1.38, and 1.40, respectively. The interference spectra of the Cargille-liquids with different RI also shift to a longer wavelength region as the T increases. When the liquid’s RI is close to the RI of cladding, it would obtain a much high T-sensitivity result, as shown in Figure 6e. It also can be understood by the theoretical T-sensitivity estimated by Equation (6). The denominator term $n_{\mathrm{cl}}^{\mathrm{eff}}$ (T) −${\;n}_{\mathrm{co}}^{\mathrm{eff}}$ (T) is getting small as the $n_{\mathrm{co}}^{\mathrm{eff}}$ (T) is close to $n_{\mathrm{cl}}^{\mathrm{eff}}$ (T), to make the T-sensitivity substantially increase. The analyzed results are very close to our experimental measurement. The above experimental data also estimated the TOCs of the Cargille-liquids with different n_D_. The obtained results were compared with those of the literature and they are organized in Table 2. It can be noticed that the measured TOCs are very close to the reference data in the literature.

**Figure 7:**
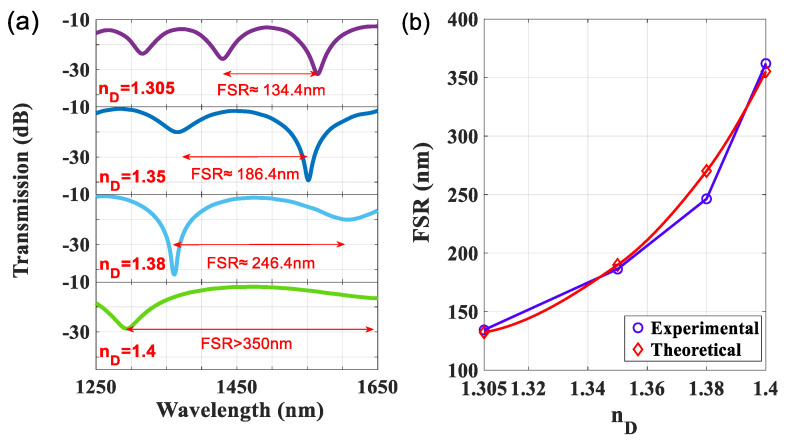
(**a**) Measured transmission spectra for LLCFMZI filled with Cargille-liquid with different n_D_ = 1.305, 1.35, 1.38, 1.40. (**b**) Comparison of FSR for theoretical and experimental values.

Furthermore, the FSR gets broader when the filling liquid has a higher RI, as plotted in Figure 7a. It is also estimated by Equation (3), since the $n_{\mathrm{cl}}^{\mathrm{eff}}\;$>$\;n_{\mathrm{co}}^{\mathrm{eff}}$, the optical path of liquid-core ($n_{\mathrm{co}}^{\mathrm{eff}}$·L) approach to $n_{\mathrm{cl}}^{\mathrm{eff}}$·L can get a very high FSR. The FSR can be high up to over 350 nm by filling the Cargille-liquids of n_D_ = 1.4. The comparison of the FSR values of the theoretical (by Equation (3)) and experimental data is shown in Figure 7b to indicate the agreement.

**Figure 8:**
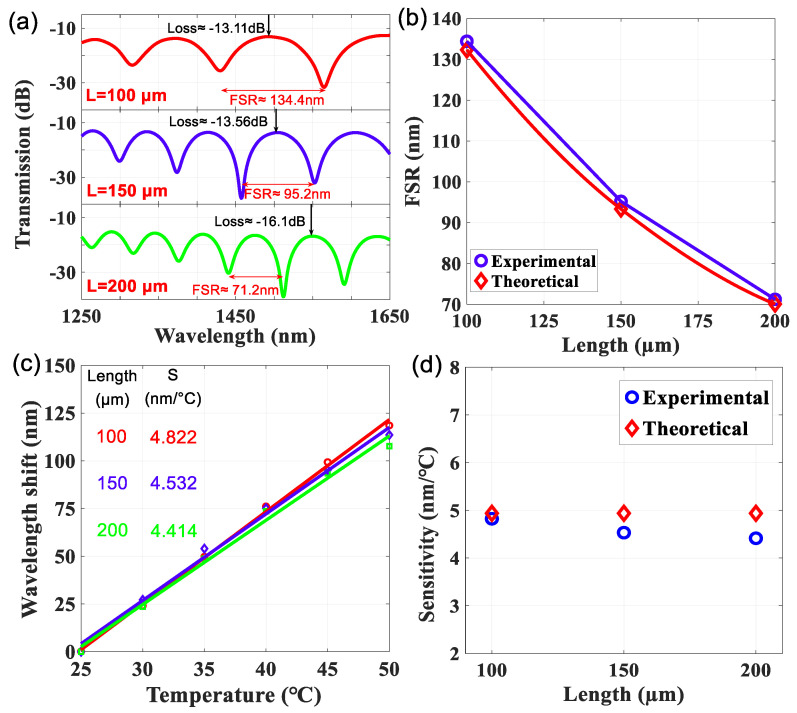
(**a**) Interference spectra, (**b**) theoretical and experimental results of the FSR, (**c**) experimental T-sensitivity, and (**d**) experimental and theoretical T-sensitivity by introducing a Cargille-liquid ($n_{D}\;$= 1.305) into the HCF with L~100, 150, and 200 μm.

By using the technique of cleaving HCF, the LLCFMZIs with different L of HCF were fabricated. They were filled with the same liquid of Cargille-liquid ($n_{D}$ = 1.305) and their spectral interference fringes, as well as optical characteristics, were measured, as shown in Figure 8. The FSRs are 134.4, 95.2, and 71.2 nm, corresponding to the HCF of ~100, 150, and 200 μm, respectively. It can be observed from Figure 8a that optical light leaks with a larger energy loss in the transmission spectra as the L becomes longer. The insertion losses are −13.11, −13.56, and −16.1 for the cases of L = 100, 150, and 200 μm, respectively.

It can be seen from Equation (3) that the length of the liquid core is inversely proportional to the FSR, which makes FSR become narrower in the spectra when the L increases. Figure 8b shows the comparison of the FSR for theoretical and experimental values in the LLCFMZIs with different L. Afterward, these LLCFMZIs were measured for the evaluation of the thermal effect. They were heated from 25 to 50 °C and the interference spectra were recorded and analyzed. The wavelengths of experimental spectra still shift toward longer wavelengths and their thermal responses of Δλ are illustrated in Figure 8c. It is worth observing that the T-sensitivities are approximately comparable. The experimental T-sensitivities for the LLCFMZIs with L of 100, 150, and 200 μm are 4.822, 4.532, and 4.414 nm/°C, respectively. The theoretical T-sensitivity was estimated as 4.938 nm/°C by Equation (6). The experimental result is consistent with that of the theoretical and the T-sensitivity is almost unaffected by the length of the liquid core. However, we found that there is a slight difference in the T-sensitivity of different lengths, as displayed in Figure 8c. We think that would be the measured wavelength (λ) of interference dip not always fixed at 1550 nm. According to Equation (6), the T-sensitivity (S) is mainly affected by the λ when the parameters of materials $k_{\mathrm{co}},k_{\mathrm{cl}},n_{\mathrm{cl}}^{\mathrm{eff}}\left(T\right),{\;\mathrm{and}\;n}_{\mathrm{co}}^{\mathrm{eff}}\left(T\right)$ are fixed. Figure 8d shows the comparison of T-sensitivity for theoretical and experimental values in the LLCFMZIs with different L.

## 4. Conclusions

We proposed a novel, ultracompact leaky-guided liquid-core fiber Mach–Zehnder interferometer (LLCFMZI) for achieving high sensitivity and the measurement of the thermal optics coefficient (TOC) of materials. The structure of the LLCFMZI is based on a tiny section of the hollow-core fiber (HCF) splicing a tilt endface single-mode fiber (SMF) to create a miniature gap for liquid access. The liquid in the hollow-core has a relatively lower refractive index (RI) than that of the cladding. Thus, a leaky mode optical waveguide (LMOW) is stimulated in the transmission. Using a micron-sized HCF is necessary to generate the MZ interference for the comparable light energy of the core and cladding modes before the light completely dissipates and disappears in the leaky-guided liquid-core. Moreover, the liquid-core with high TOC and dispersion can achieve highly modulated spectra and excellent T-sensitivity performances. The T-sensitivity of the LLCFMZI is extremely influenced by the TOC of the $n_{\mathrm{co}}^{\mathrm{eff}}\left(T\right)$ of the core mode. Experimental results confirmed that the interference spectra of the LLCFMZI can achieve very high spectral shifts within a wide range (over 1250–1650 nm) and with a steadily linear response for the thermal variations. Given that the RI of liquids is highly related to T, the TOC of the materials can be also determined by analyzing the measured spectral responses. The measured T-sensitivities of the DI water, ethanol, and Cargille-liquid (n_D_ = 1.305) are 0.8869, 4.4754, and 4.8229 nm/°C, respectively, and their corresponding TOCs are −4.16 × 10^−5^, −2.11 × 10^−4^, and −3.6 × 10^−4^ °C^−1^. The highest sensitivity of the liquid core filled with Cargille-liquid (n_D_ = 1.40) reaches 13.87 nm/°C and the corresponding TOC −4.07 × 10^−4^ °C^−1^ is measured. Regarding the quality of interference, fringe visibility that exceeds 30 dB can be obtained in the structure. Note that the proposed LLCFMZI based on the leaky-guided liquid-core waveguide with only a few picoliters of liquids in the HCF can achieve an ultracompact, stable, highly sensitive, and accurate TOC measurement.

## Figures and Tables

**Table 1:** Comparisons of the TOCs for three types of liquids between the experimental results proposed by this study and that of the reference data.

Liquids	DI Water (at 1550 nm)	Ethanol (at 1550 nm)	Cargille-Liquid (n_D_ = 1.305)
TOC: k_1_ (°C^−1^) (measured in the study)	−4.16 × 10^−5^	−2.11 × 10^−4^	−3.60 × 10^−4^ (at 1550 nm)
TOC: k_1_ (°C^−1^) (reference data)	−7.657 × 10^−5^ [33]	−3.688 × 10^−4^ [33]	−3.34 × 10^−4^ (at 589 nm) [32]
	−8 × 10^−5^ [34]	−2.59 × 10^−4^ [35]	

**Table 2:** Comparisons of TOCs for different Cargille-liquids between the experimental results and that of the reference data.

Cargille-Liquids	n_D_ = 1.305	n_D_ = 1.35	n_D_ = 1.38	n_D_ = 1.40
TOC: k_1_ (°C^−1^) (at 1550 nm) (measured in the study)	−3.60 × 10^−4^	−3.35 × 10^−4^	−2.76 × 10^−4^	−4.07 × 10^−4^
TOC: k_1_ (°C^−1^) (at 589 nm) (reference data)	−3.34 × 10^−4^ [32]	−3.40 × 10^−4^ [32]	−3.43 × 10^−4^ [32]	−4.12 × 10^−4^ [32]
